# A Randomized Controlled Study on the Effects of Bisoprolol and Atenolol on Sympathetic Nervous Activity and Central Aortic Pressure in Patients with Essential Hypertension

**DOI:** 10.1371/journal.pone.0072102

**Published:** 2013-09-10

**Authors:** Wei-Jun Zhou, Ren-Ying Wang, Yan Li, Dong-Rui Chen, Er-Zhen Chen, Ding-Liang Zhu, Ping-Jin Gao

**Affiliations:** 1 State Key Laboratory of Medical Genomics, Shanghai Key Laboratory of Hypertension and Department of Hypertension, Ruijin Hospital, Shanghai Jiao Tong University School of Medicine, Shanghai, China; 2 Shanghai Institute of Hypertension, Shanghai, China; 3 Laboratory of Vascular Biology, Institute of Health Sciences, Shanghai Institutes for Biological Sciences, Chinese Academy of Sciences, Shanghai, China; 4 Department of Emergency, Ruijin Hospital, Shanghai Jiao Tong University School of Medicine, Shanghai, China; College of Pharmacy, University of Florida, United States of America

## Abstract

**Objective:**

β-blockers (BBs) with different pharmacological properties may have heterogeneous effects on sympathetic nervous activity (SNA) and central aortic pressure (CAP), which are independent cardiovascular factors for hypertension. Hence, we analyzed the effects of bisoprolol and atenolol on SNA and CAP in hypertensive patients.

**Methods:**

This was a prospective, randomized, controlled study in 109 never-treated hypertensive subjects randomized to bisoprolol (5 mg) or atenolol (50 mg) for 4–8 weeks. SNA, baroreflex sensitivity (BRS) and heart rate (HR) variability (HRV) were measured using power spectral analysis using a Finometer. CAP and related parameters were determined using the SphygmoCor device (pulse wave analysis).

**Results:**

Both drugs were similarly effective in reducing brachial BP. However, central systolic BP (−14±10 mm Hg vs −6±9 mm Hg; *P*<0.001) and aortic pulse pressure (−3±10 mm Hg vs +3±8 mm Hg; *P*<0.001) decreased more significantly with bisoprolol than with atenolol. The augmentation index at a HR of 75 bpm (AIxatHR75) was significantly decreased (29%±11% to 25%±12%; *P* = 0.026) in the bisoprolol group only. Furthermore, the change in BRS in the bisoprolol group (3.99±4.19 ms/mmHg) was higher than in the atenolol group (2.66±3.78 ms/mmHg), although not statistically significant (*P*>0.05). BRS was stable when RHR was controlled (RHR≤65 bpm), and the two treatments had similar effects on the low frequency/high frequency (HF) ratio and on HF.

**Conclusion:**

BBs seem to have different effects on arterial distensibility and compliance in hypertensive subjects. Compared with atenolol, bisoprolol may have a better effect on CAP.

**Trial Registration:**

ClinicalTrials.gov NCT01762436

## Introduction

The sympathetic nervous system (SNS) plays a role in the pathophysiology of chronic arterial hypertension by modifying cardiac output and peripheral vascular resistance [1], [2]. It is known that sympathetic nerve activity (SNA) can cause changes in blood pressure (BP) through the activation of baroreceptors [3], [4]. Although introduced into scientific practice, methods for SNA evaluation are not commonly used in a clinical setting. Analysis of baroreflex sensitivity (BRS) and heart rate (HR) variability (HRV) have been recommended as the diagnostic tools for evaluating SNA, and can be found in clinical guidelines as basic assessment methods [5]–[7]. Data suggest that low BRS and/or HRV are risk factors for cardiovascular morbidity and mortality [8]–[10].

Increasing clinical evidence suggests that central aortic pressure (CAP), but not brachial BP, predicts cardiovascular events, because the left ventricle (LV) pumps directly against the afterload in the central arteries. Moreover, aortic systolic BP, pulse pressure (PP), and augmentation index (AIx) have been shown to be strong independent cardiovascular risk factors in hypertensive populations [11]–[15].

β-blockers (BBs) are believed to improve SNS function. However, clinical studies on the effects of BBs on HRV and/or BRS in hypertensive patients have shown mixed results [16], [17]. Moreover, in a number of studies [14], [18]–[22], atenolol-based therapy was significantly less effective for lowering aortic systolic and pulse pressure, which may be attributed to a different mechanism of atenolol, thus explaining the different clinical outcomes. Since β-blocking drugs might have heterogeneous effects on the arterial system and BRS depending on their pharmacologic properties, further comparisons of the effects of BBs on the arterial system and BRS may be helpful [23]. Bisoprolol, with its high β1-selectivity, long duration of action, and favorable pharmacokinetic properties, was shown to be an effective and safe antihypertensive agent [24], [25]. Supposedly, these properties of bisoprolol should be an advantage in clinical practice.

Therefore, the present study was designed to compare the effects of a highly selective β1-blocker (bisoprolol) and a classical BB (atenolol) on SNA and CAP in hypertensive patients with a controlled heart rate.

## Subjects and Methods

The protocol for this trial and supporting CONSORT checklist are available as supporting information; see Checklist S1 and Protocol S1.

### Study subjects

The study participants, aged 25–65 years with never-treated mild-to-moderate essential hypertension (EH), with normal sinus rhythm and a resting HR (RHR) of >70 bpm, were recruited from the hypertension clinic at the Ruijin Hospital, Shanghai, between October 2010 and March 2012. Mild-to-moderate EH was defined as a systolic BP of 140–160 mmHg and/or a diastolic BP of 90–100 mmHg on at least three different occasions separated by a month. Subjects with secondary hypertension, diabetes mellitus (DM), bradyarrhythmia/hypotension, bronchial asthma, or liver dysfunction/renal impairment were excluded (please see the online Data Supplement at http://clinicaltrials.gov/ct2/show/NCT01762436). Experimental protocol and informed consent were approved by the ethics committee of the Ruijin Hospital, Shanghai Jiaotong University (approval ID [2012]36), and informed consent to participate in the study was provided by the patients or their relatives. All patients signed their informed consent.

### Study design

This was a prospective, two-center, open label, parallel, randomized controlled study, focusing on SNA (registered at Clinicaltrial.Gov; NCT01251146; http://clinicaltrials.gov/ct2/show/NCT01251146). In our hospital, one of the two centers of the main trial, a substudy on central blood pressure was conducted (registered at Clinicaltrial.Gov; NCT01762436; http://clinicaltrials.gov/ct2/show/NCT01762436). This present article describes this substudy. Both the main trial and the substudy were approved by the Ethics Committee of the Ruijin Hospital, Shanghai Jiaotong University. All patients were randomized to bisoprolol (group A) or atenolol (group B) in a 1∶1 ratio using a predesigned randomization schedule, stratified by study center. Sealed envelopes were used for assigning patients to their treatment. The sample size calculation and randomization table were performed using SAS® v9.3 (SAS Institute, North Carolina, USA).

**Figure 1:**
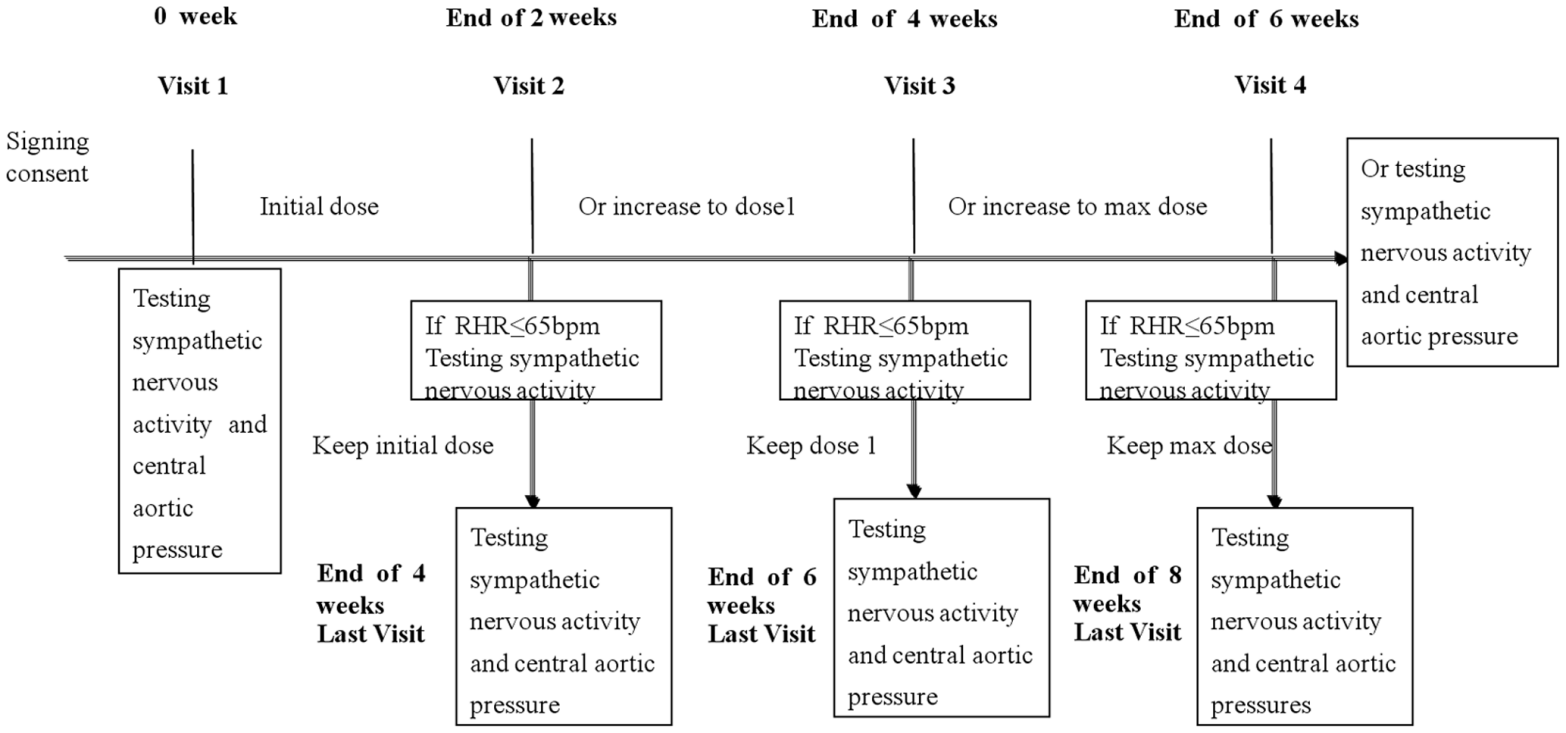
Study schedule. RHR: resting heart rate.

Subjects in group A initially received 5 mg of bisoprolol (Concor®, Merck Serono, Darmstadt, Germany), and those in group B received 50 mg of atenolol (Beijing Double-Crane Pharmaceutical Co., Ltd, Beijing, China), once daily. RHR was assessed every two weeks. If the RHR was ≤65 bpm, a 2-week maintenance treatment was added during the final visit. If the target RHR was not achieved, the dose was changed as recommended in the study protocol. The maximal dose was 10 mg qd for bisoprolol and 100 mg qd for atenolol. The longest treatment period was 8 weeks. If the patient's RHR did not reach <65 bpm at week 6, the treatment was ended at week 6 (Figure 1).

Detailed patient information pertaining to the hypertension and cardiovascular history, hypercholesterolemia, DM, alcohol consumption, and smoking was obtained from medical records. Smoking status of the patients was defined as smokers and nonsmokers.

### Measurements

During physical examination, age, gender, body weight, body mass index (BMI), and abdomen circumference of the patients were recorded. Echocardiography (Philips IE33 system, Philips Medical Systems, Bothell, WA, USA) was also performed.

**Brachial BP measurements** at 5-min intervals were carried out 3 times using a semiautomated oscillometric device (Omron HEM-7011; Omron Healthcare, Dalian, China). The mean of the last two measurements was used in the analyses. The non-dominant arm was used for all BP measurements.

**CAP analysis** was performed by pressure tonometry using the integrated software (SphygmoCor; AtCor Medical, Sydney, Australia) of the radial pulse, considering that this system has shown good repeatability of measurements [26]–[28]. AIx, a measure of systemic arterial stiffness [27], was calculated as the difference between the second and first systolic peaks, expressed as a percentage of the pulse pressure. Because AIx depends on HR, it was corrected for a HR of 75 bpm (AIxatHR75) [29]. Mean arterial pressure was calculated by the integration of the radial artery waveform. The degree of PP amplification was calculated as brachial PP/central PP.

Measurements of SNA were performed after a 30-min rest in the sitting position. Patients were monitored non-invasively with a Finometer (Finapres Medical Systems, Amsterdam, The Netherlands; factory number: FMI. MU 00694, 00637; operation parameters: input: 220–240 V, 50–60 Hz, 100 VA) in the supine and the standing positions. BRS was defined by the slope of the linear regression curve obtained by plotting the changes of SBP against the pulse interval. The mean value of the various slopes was calculated and used as the definitive BRS value for each subject [5], [30]–[33]. HRV was calculated from the time-sequential analysis and was expressed as three components: low frequency (LF), high frequency (HF), and LF/HF ratio. Additionally, blood pressure variability (BPV) was calculated.

RHR was measured by 12-lead electrocardiography [34] in the supine position.

### Procedures at follow-up

Follow-up visits were scheduled every 2 weeks. At each visit, a clinical evaluation was performed as per study protocol, which included recording the BP at approximately the same time of the day, and measuring RHR in duplicate by the same person for each individual subject. CAP and SNA evaluation were carried out at baseline and at the final visit. An additional SNA test was performed at week 6 when the subjects may or may not have achieved target RHR (Figure 1). All measurements were performed by physicians who were blinded to the treatment, clinical data, and physical examination. In addition to a full clinical assessment, ancillary examinations were performed at the beginning and at the end of the final visit, including a complete blood count, hepatic and renal function tests, blood glucose and total serum cholesterol measurements, urinalysis, and electrocardiography. Patient compliance was confirmed at each visit by capsule counting. Adverse events were monitored throughout the study and recorded at each visit. Data were then reviewed by an independent medical committee.

### Data analysis

The primary outcome was the change in CAP. Secondary outcomes were changes in BRS, HRV, and peripheral BP. SAS 9.2 (SAS Institute Inc., Cary, NC, USA) was used for data analysis, on an intention-to-treat (ITT) basis. Continuous variables are presented as mean ± standard deviation (SD), while categorical variables are presented as proportions. In each group, comparisons between baseline and variations were made using two-tailed Student's *t*-test for paired observations. Independent samples *t*-test was used to compare normally distributed continuous variables between the two groups, while the Wilcoxon rank-sum test was used to analyze normal distributed continuous data. Chi-squared test (χ^2^) and Fisher's exact test were used to compare proportions. Comparisons of hemodynamic parameters (brachial BP and CAP) were performed using analysis of covariance (ANCOVA) and were adjusted for age, gender, and BMI.

## Results

### Clinical characteristics of study subjects

A total of 126 patients with hypertension were enrolled in the study. Seventeen patients were withdrawn for various reasons stated in the exclusion criteria. A total of 11 patients (8 patients in the atenolol group and 3 patients in the bisoprolol group) were lost to follow-up, resulting in 109 patients for the final ITT analysis.

Patients were randomly assigned to two groups: the bisoprolol group (54 patients, 37 (68.52%) men, mean age of 43 years); and the atenolol group (55 patients, 38 (69.09%) men, mean age of 44 years). Subjects' baseline characteristics are summarized in Table 1. As expected, there were no significant differences in age, gender, BMI, echocardiographic parameters, blood glucose, and total serum cholesterol levels between the two groups (*P*>0.05).

**Table 1:** Clinical characteristics of the study population (ITT).

Measurements	Bisoprolol (n = 54)	Atenolol (n = 55)
Age (y)	43.11±9.80	44.76±10.99
Gender, male (%)	37 (68.52)	38 (69.09)
Current smoker (%)	17 (31.48)	19 (34.55)
BMI (kg/m^2^)	24.59±3.03	24.91±3.63
Abdomen circumference (cm)	87.87±10.80	88.98±11.00
Serum glucose (mmol/L)	5.51±0.85	5.37±0.65
Triglycerides (mmol/L)	1.82±1.27	1.89±1.81
Total cholesterol (mmol/L)	5.05±0.80	4.85±1.07
HDL cholesterol (mmol/L)	1.33±0.37	1.24±0.31
LDL cholesterol (mmol/L)	3.13±0.79	2.97±0.98
Serum urea nitrogen (mmol/L)	4.41±1.18	4.51±1.21
Serum creatinine (mmol/L)	73.89±15.09	71.71±14.99
Serum sodium (mmol/L)	138.69±1.92	138.96±2.05
Serum potassium(mmol/L)	4.13±0.28	4.18±0.37
Serum chlorine(mmol/L)	104.79±2.31	105.14±2.51
LVEDD (mm)	47.73±3.74	49.11±3.96
LVESD (mm)	30.07±3.17	31.12±2.86
LVEF (%)	66.78±4.23	65.71±4.58

Data are shown as mean±SD or proportions.

BMI: body mass index; HDL: high-density lipoprotein; LDL: low-density lipoprotein; LVEDD: left ventricular end-diastolic diameter; LVEF: left ventricular ejection fraction; LVESD: left ventricular end-systolic diameter. *P*>0.05, independent-samples *t*-test of two groups or χ^2^ test; all *P*-values>0.05.

### Effects of bisoprolol and atenolol on brachial BP and CAP

BP levels were not different between the two groups before treatment, and brachial BP was significantly reduced to a similar extent after treatment in the two groups (Table 2).

**Table 2:** Comparison of changes in hemodynamic variables from baseline to the end of treatment in the bisoprolol and atenolol groups (ITT).

Parameter		Bisoprolol (n = 54)	Atenolol(n = 55)	*P*-Value
Brachial SBP (mm Hg)	Baseline	145.43±6.03	145.29±5.42	0.902
	Last visit	124.28±11.95^d^	123.64±10.62^d^	0.768
Brachial DBP (mmHg)	Baseline	91.17±7.78	89.80±7.71	0.359
	Last visit	80.69±9.18^d^	80.76±7.19^d^	0.966
Brachial MAP (mmHg)	Baseline	109.25±6.15	108.30±5.76	0.404
	Last visit	95.22±9.65^d^	95.05±7.85^d^	0.924
Brachial PP (mm Hg)	Baseline	54.26±8.12	55.49±8.56	0.443
	Last visit	43.59±6.90^d^	42.87±6.85^d^	0.586
HR(beats per minute)	Baseline	83.65±7.42	81.85±6.58	0.184
	Last visit	64.00±4.53	63.33±4.66^d^	0.446
Aortic SBP (mm Hg)	Baseline	129.94±10.57	129.13±12.81	0.717
	Last visit	116.15±12.54^d^	122.71±12.18^d^	0.007
Aortic DBP (mm Hg)	Baseline	90.76±8.03	91.00±8.16	0.877
	Last visit	80.41±8.92^d^	80.85±6.05^d^	0.760
Aortic PP (mm Hg)	Baseline	39.19±7.73	38.13±6.56	0.443
	Last visit	36.20±10.87^b^	41.84±10.36^c^	0.007
PP amplification	Baseline	1.44±0.38	1.52±0.36	0.407
	Last visit	1.30±0.45^b^	1.07±0.25^d^	0.001
AP (mm Hg)	Baseline	10.33±5.03	10.11±6.10	0.834
	Last visit	10.93±5.26^a^	13.77±6.38^d^	0.013
AIx (%)	Baseline	25.77±11.79	25.29±14.32	0.848
	Last visit	29.61±13.03^b^	32.77±13.47^d^	0.216
AIxatHR75 (%)	Baseline	29.23±11.71	28.03±14.82	0.640
	Last visit	25.21±12.62^b^	28.10±13.03^a^	0.242
BPV(mmHg^2^)	Baseline	7.55±3.95	6.96±3.38	0.414
	Last visit	6.25±2.21^a^	6.72±5.59^a^	0.577

Data are shown as mean±SD. SBP: systolic blood pressure; DBP: diastolic blood pressure; PP: pulse pressure; MAP: mean arterial pressure; AP: augmentation pressure AIx: augmentation index; BPV: blood pressure variability.

a *P*>0.05,

b *P*<0.05,

c *P*<0.01,

d *P*<0.0001; ANOVA.

**Figure 2:**
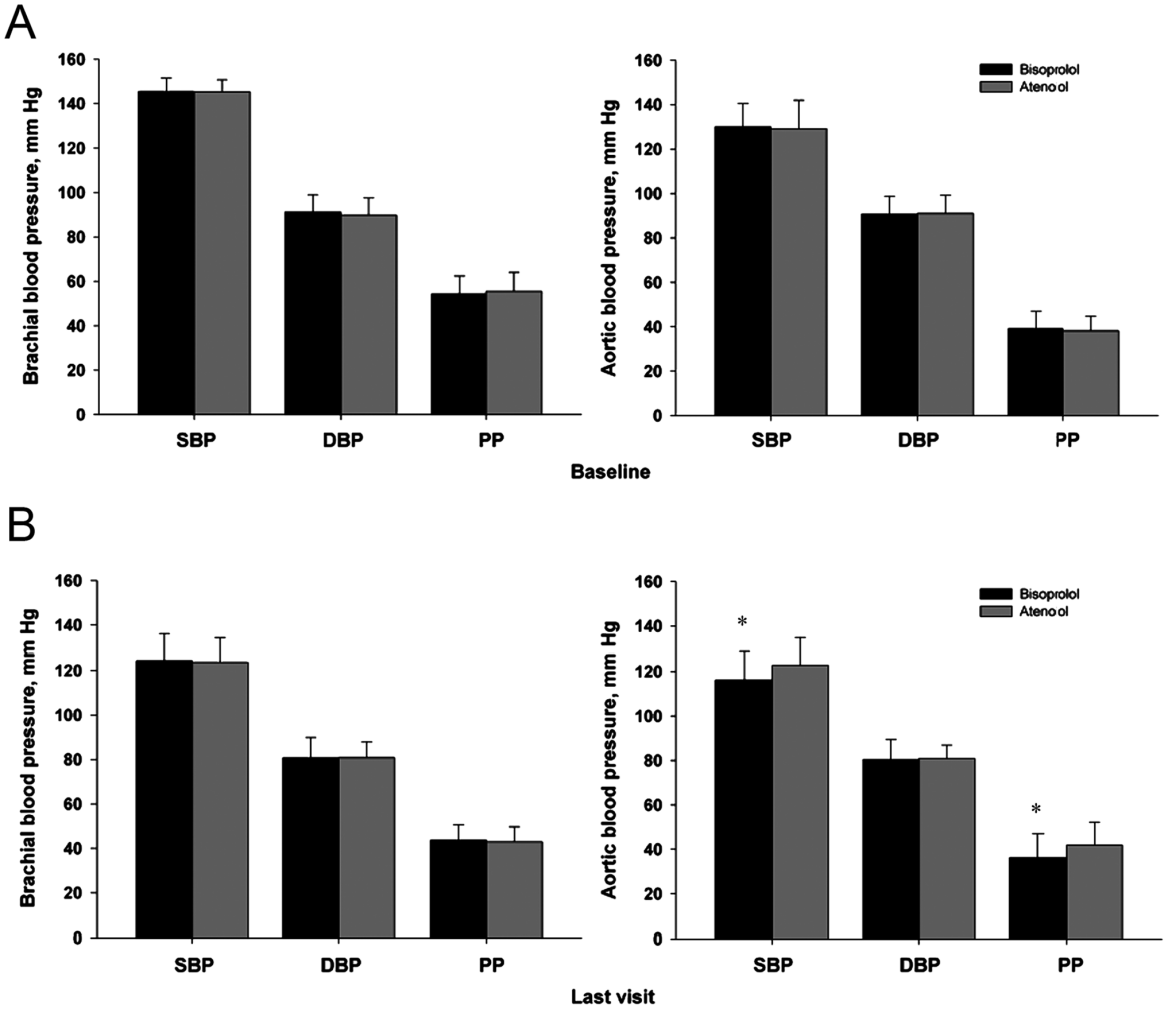
(A) Comparison of hemodynamic parameters of study subjects at baseline. (B) Comparison of hemodynamic parameters of study subjects at the end of the treatment period.*P<0.01.

To compare the effect of bisoprolol and atenolol on CAP, radial arterial waveforms were recorded, and the corresponding aortic waveforms were generated, from which aortic systolic BP (SBP) and diastolic BP (DBP) were calculated. As shown in Table 2, the decrease in aortic SBP was significantly higher in the bisoprolol group than in the atenolol group (*P* = 0.007). Aortic PP and augmentation pressure (AP) significantly increased in the atenolol group (*P*<0.05), and the PP amplification significantly decreased in the atenolol group compared with the bisoprolol group (*P* = 0.001). The AIxatHR75 value was significantly decreased (29±12% to 25±13%; *P* = 0.026) in the bisoprolol group only, which seemed to be independent from HR. There was no significant change in BPV by the end of the treatment period in the two groups (Table 2, Figure 2).

### Effects on SNA in the two groups

To assess the efficiency of the two BBs on SNA, assessment of BRS and HRV was performed using a Finometer device. Results showed that the change in BRS in the bisoprolol group (3.99±4.19 ms/mmHg) was higher than in the atenolol group (2.66±3.78 ms/mmHg) at the final visit, but the difference was not statistically significant (*P* = 0.107). Furthermore, there was no difference in BRS between the final visit and the visit during which the target HR was achieved. These results suggest that RHR was correlated with BRS (Table 3).

**Table 3:** Changes in BRS from baseline to the end of treatment in the bisoprolol and atenolol groups (ITT).

BRS	Bisoprolol (n = 54)	Atenolol (n = 55)	*P*-Value
Baseline	8.02±2.78	8.22±3.39	0.748
HR target achieved visit*	11.56±5.34	10.41±4.63	0.275
Δ baseline–HR target achieved visit*	3.24±3.96	1.97±4.01	0.134
*P*-value	<.0001	0.002	
Last visit	12.25±5.31	11.13±4.71	0.269
ΔLast visit- baseline	3.99±4.19	2.66±3.78	0.107
*P*-value	<.0001	<.0001	
Δ Last visit– HR target achieved visit*	0.79±4.81	0.97±3.57	0.837
*P*-Value	0.268	0.082	

* Visit (week 2, 4 or 6) at which patients achieved the RHR target of <65 bpm.

**Figure 3:**
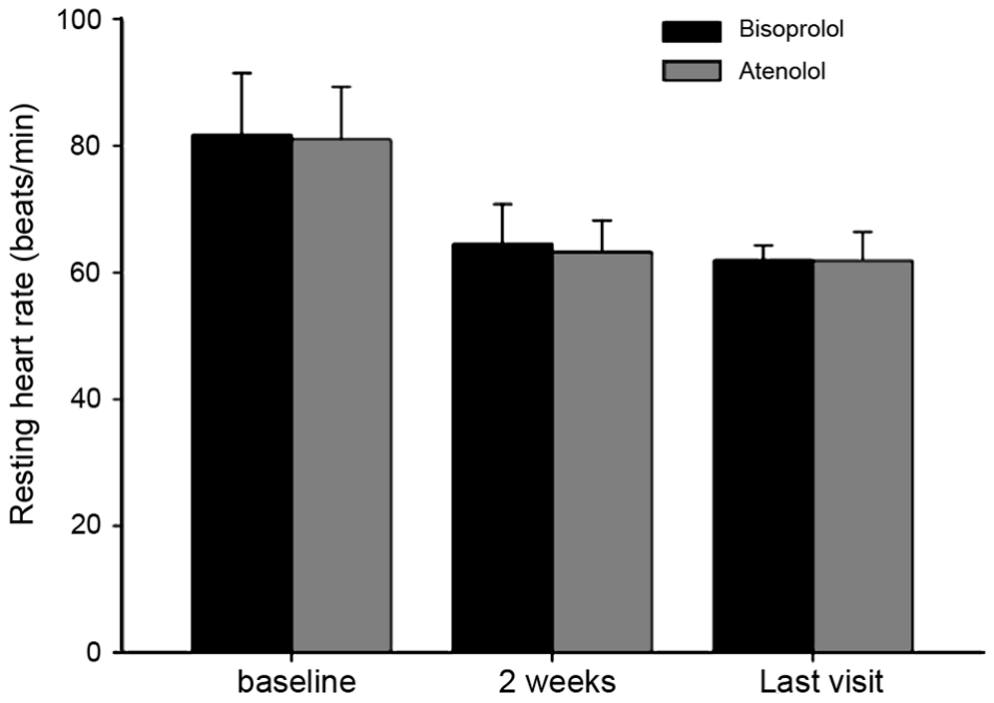
Changes in RHR from baseline to the end of treatment in the bisoprolol and atenolol groups.

HRV was evaluated by the measurement of three components: LF, HF, and LF/HF ratio. LF was significantly increased in the bisoprolol group compared with baseline. However, LF in the atenolol group showed a non-significant change at the end of treatment. The two treatments had similar effects on the mean change in HF and LF/HF ratio from baseline (Table 4). RHR decreased significantly from baseline in each treatment group, being almost identical between the two drugs (Figure 3).

**Table 4:** Changes in HRV from baseline to the end of treatment in the bisoprolol and atenolol groups (ITT).

Parameter		Bisoprolol (n = 54)	Atenolol (n = 55)	*P*-Value
LF (ms^2^)	**Baseline**	254.63±187.69	318.31±333.76	0.229
	**Last visit**	328.93±218.93^b^	311.04±297.87^a^	0.732
HF (ms^2^)	**Baseline**	188.24±175.80	215.55±250.78	0.518
	**Last visit**	379.86±373.50^d^	388.82±495.55^b^	0.919
LF/HF	**Baseline**	1.94±1.62	2.47±2.30	0.173
	**Last visit**	1.30±1.14^c^	1.36±1.30^d^	0.818

Data are shown as mean±SD. HF: high frequency; HRV: heart rate variability; LF: low frequency;

a *P*>0.05,

b *P*<0.05,

c *P*<0.01,

d *P*<0.0001.

### Safety profile

No serious adverse events were reported in either of the two treatment groups. No patient was withdrawn from the study because of adverse events. No significant changes in biochemical parameters were observed after treatment (*P*>0.05) (data not shown).

## Discussion

In the present study, we performed a comparative analysis to evaluate the effects of two antihypertensive drugs, namely bisoprolol and atenolol, on SNA and CAP. Brachial BP was reduced to a similar extent in both groups, whereas bisoprolol had a more marked effect on aortic SBP and PP, and caused more important decreases in these parameters than atenolol (*P* = 0.007). Our study represents the first available evidence that bisoprolol has a beneficial effect on peripheral vascular resistance and is relatively effective in lowering central SBP.

Our study also revealed that although there was an increase in aortic AIx with the use of both BBs, the AIxatHR75 (a measure of systemic arterial stiffness at an HR of 75 bpm) significantly decreased in the bisoprolol group, whereas no change in AIxatHR75 was observed in the atenolol group. AIx was found to be very strongly correlated with changes in HR [29], which can explain these different results between the two groups. Because this correlation was significant only under bisoprolol treatment, it may suggest a more important central reduction in BP and a reduction in peripheral vascular resistance rather than a change in HR. Moreover, the correlation seems independent of HR for CAP.

Three factors can explain the reasons for the more important decrease in CAP with bisoprolol: first, because of its high selectivity towards β1 adrenergic receptors vs. β2 receptors (at doses up to 10 mg, 0–5% of β2 receptors are blocked), bisoprolol blocks a lower number of β2 receptors than does atenolol (at a daily dose of 100 mg, approximately 25% of β2 receptors are blocked) [23]. It is reported that β2 stimulation causes vasodilatation, benefiting arterial elasticity, and lowers CAP [35]; β2 blockade would antagonize this potentially beneficial process. Second, the decrease in central SBP reflects a significant improvement in the function of the large arteries and a changed pattern of the peripheral reflection coefficients as well as the structural arterial network. Bisoprolol, along with improving the viscoelastic properties of the arterial wall, has been found to decrease arterial pulse wave velocity (PWV) and to increase arterial compliance in hypertensive patients or normotensive subjects [36]–[38]. A study confirmed that atenolol does not improve vascular compliance [39], and that atenolol is relatively ineffective in lowering central aortic systolic pressure [14], [18]–[22]. Third, it was reported that bisoprolol failed to potentiate the constrictor response to noradrenaline, and that it antagonized the constrictor responses both to noradrenaline and the selective α1-adrenoceptor agonist, PE [40], [41]. In addition, another study in rats showed that bisoprolol treatment lowered the production of the vasoconstrictive endothelin-1 (ET-1) and thromboxane [42]. These results suggest that bisoprolol, which is able to lower epinephrine and renin activity [43], may also dilate the vessels and markedly lower central SBP, similar to the action of another selective β-1-adrenoceptor antagonist, nebivolol [44]. Our results showed that bisoprolol, but not atenolol, could induce vasorelaxation of rats aorta rings. Bisoprolol's vasodilating effects depend on endothelium-dependent mechanisms, as inferred from their attenuation by nitric oxide synthase (NOS) inhibitors (L-Nω-nitroarginine methyl ester (L-NAME)) (see File S1. Effects of bisoprolol and atenolol on aortic vasorelaxation in rats.). Thus, BBs seem to have dissimilar effects on arterial distensibility and compliance in subjects with elevated blood pressure.

To date, only a few studies compared the effects of BBs on BRS and HRV. These studies vary considerably in their design and in their methodologies to measure HRV and BRS, thus making it difficult to compare different antihypertensive agents in terms of their effect on SNA [17]. In the present study, we used a simple and precise method for the measurement of finger arterial pressure using the Beatscope software, which has been previously used to accurately assess the effects of BBs on SNA in patients with essential hypertension [5]. As expected, our findings are in concordance with those of previous studies in spontaneously hypertensive rats [45], [46], demonstrating that bisoprolol or atenolol not only exert a depressor action in hypertension, but also improve abnormal baroreflex function associated with a marked decrease in the lower HR plateau. These effects are consistent with the pharmacodynamic properties of the two drugs [47], [48].

In addition, there are some important issues that deserve to be mentioned. Although the two study drugs have different pharmacological characteristics, no significant differences in BRS were found between the two groups at the end of the treatment period, which was contrary to our expectation. A possible explanation for this result could be that the effects of BBs on BRS have been consistent with their RHR-lowering action. It is clear that the majority of the benefit of β1 adrenergic receptor blockade is mediated via a decrease in HR [49]. Our results showed that the increase in BRS and HF was similar between the two treatment groups. Thus, antihypertensive therapy, which effectively increases BRS, should have a desirable effect on HRV.

Limitations of our study are the small sample size and the short duration of the study, which may be the reason for not being able to detect a significant difference in SNA as a result of the two different antihypertensive therapies. A future study should be designed to assess the changes in vascular function and SNA for these two BBs in a larger trial.

In conclusion, our study demonstrates that hypertension is associated with a decrease in BRS, which may be improved by antihypertensive therapy using BBs. Bisoprolol, which seems to act independently of BRS, may dilate the vessels and have a better effect on CAP than the standard β-blocker comparator, atenolol.

## Supporting Information

Abbreviations S1 Abbreviations and Acronyms.
(DOC)

Checklist S1 CONSORT Checklist
(DOC)

CONSORT Diagram S1 Patient Flow Diagram.
(DOC)

Figure S1 Effects of bisoprolol and atenolol on aortic vasorelaxation in rats.
(TIF)

File S1 Effects of bisoprolol and atenolol on aortic vasorelaxation in rats.
(DOC)

Protocol S1 **Trial Protocol.**
(PDF)
