# Supplementary material for: A Randomized Controlled Study on the Effects of Bisoprolol and Atenolol on Sympathetic Nervous Activity and Central Aortic Pressure in Patients with Essential Hypertension
Source: PLoS One. 2013 Sep 10;8(9):e72102. doi: 10.1371/journal.pone.0072102 (PMC3769307; doi:10.1371/journal.pone.0072102)
Supplement: Abbreviations S1 — Abbreviations and Acronyms. (DOC) [file pone.0072102.s001.doc]

Abbreviations and Acronyms

| AE  Aix  AP | Adverse Event  Augmentation Index  Augmentation Pressure |
| --- | --- |
| ANCOVA  BBs  BMI  BP | Analysis Of Covariance  β-Blockers  Body Mass Index  Blood Pressure |
| BPV | Blood Pressure Variability |
| BRS  BUN  CAP | Baroreflex Sensitivity  Blood Urea Nitrogen  Central Aortic Pressure |
| DBP  EH  HDL | Diastolic Blood Pressure  Essential Hypertension  High Density Lipoprotein |
| HF | High Frequency |
| HRV | Heart Rate Variability |
| LF | Low Frequency |
| ITT  LDL | Intention to Treat  Low Density Lipoprotein |
| LVEDD  LVEF  LVESD  MAP | Left Ventricular End-Diastolic Diameter  Left Ventricular Ejection Fraction  Left Ventricular End-Systolic Diameter  Mean Arterial Pressure |
| PP | Pulse Pressure |
| RHR | Resting Heart Rate |
| SAE | Serious Adverse Event |
| SBP | Systolic Blood Pressure |
| SD  SNA | Standard Deviation  Sympathetic nerve activity |
| SNS | Sympathetic Nervous System |
