# Supplementary material for: A Randomized Controlled Study on the Effects of Bisoprolol and Atenolol on Sympathetic Nervous Activity and Central Aortic Pressure in Patients with Essential Hypertension
Source: PLoS One. 2013 Sep 10;8(9):e72102. doi: 10.1371/journal.pone.0072102 (PMC3769307; doi:10.1371/journal.pone.0072102)
Supplement: CONSORT Diagram S1 — Patient Flow Diagram. (DOC) [file pone.0072102.s003.doc]

**Patient Flow Diagram**

**Allocation**

**Analysis**

**Follow-Up**

**Enrollment**

Assessed for eligibility (n=126 )

Excluded (n=17 )

♦  Not meeting inclusion criteria (n=17 )

♦  Declined to participate (n=0 )

♦  Other reasons (n=0 )

Analysed (n=51)
♦ Excluded from analysis (n= 0 )

Lost to follow-up (withdraw from study) (n=3 )

Discontinued intervention (n=0 )

Allocated to intervention (n=54 )

♦ Received allocated intervention (n=54 )

♦ Did not receive allocated intervention (give reasons) (n=0 )

Lost to follow-up (withdraw from study) (n= 8 )

Discontinued intervention (n= 0 )

Allocated to intervention (n=55 )

♦ Received allocated intervention (n=55 )

♦ Did not receive allocated intervention (give reasons) (n=0 )

Analysed (n=47)
♦ Excluded from analysis (n= 0 )

Randomized (n=109 )
