# Supplementary figures and images for: A Randomized Controlled Study on the Effects of Bisoprolol and Atenolol on Sympathetic Nervous Activity and Central Aortic Pressure in Patients with Essential Hypertension
Source: PLoS One. 2013 Sep 10;8(9):e72102. doi: 10.1371/journal.pone.0072102 (PMC3769307; doi:10.1371/journal.pone.0072102)

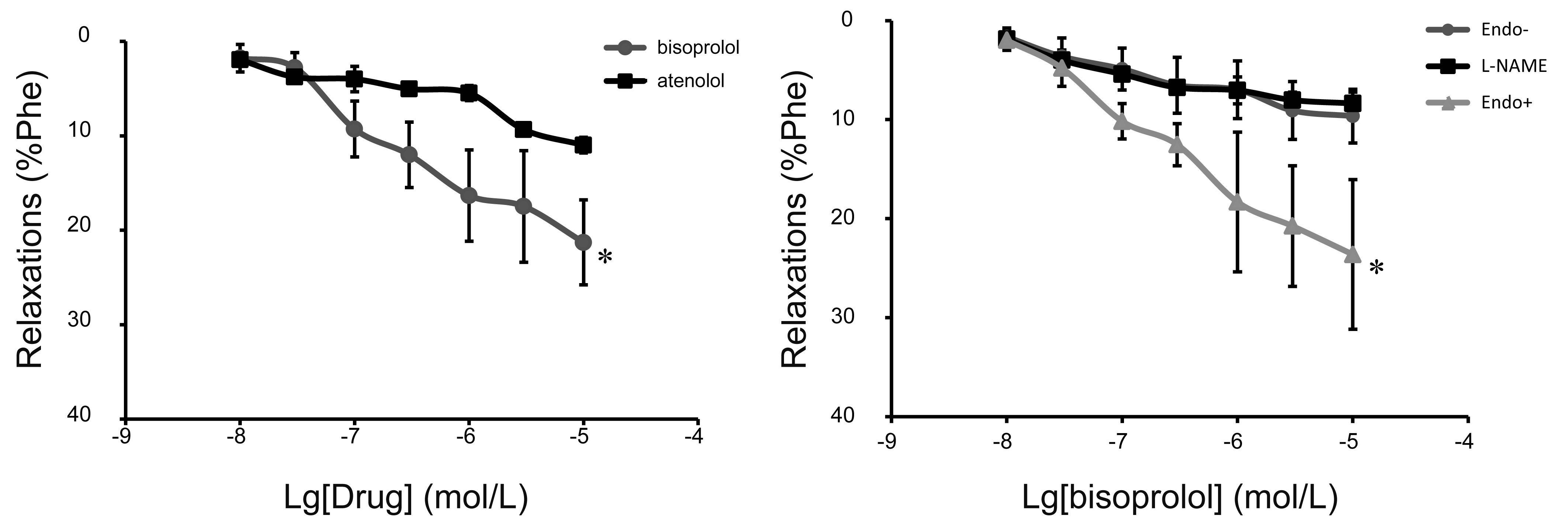

Supplement: Figure S1 — Effects of bisoprolol and atenolol on aortic vasorelaxation in rats. (TIF) [file pone.0072102.s004.tif]
