# Supplementary material for: A Randomized Controlled Study on the Effects of Bisoprolol and Atenolol on Sympathetic Nervous Activity and Central Aortic Pressure in Patients with Essential Hypertension
Source: PLoS One. 2013 Sep 10;8(9):e72102. doi: 10.1371/journal.pone.0072102 (PMC3769307; doi:10.1371/journal.pone.0072102)
Supplement: File S1 — Effects of bisoprolol and atenolol on aortic vasorelaxation in rats. (DOC) [file pone.0072102.s005.doc]

**Effects of bisoprolol and atenolol on aortic vasorelaxation in rats.**

**【Abstract】**

**Objective：** Since the impairment of β-Blocker bisoprolol induced vascular relaxation has not been described in the literature, the aim of the present study was to determine the effects of bisoprolol and atenolol on vascular reactivity in aortic rings from Sprague-Dawley (SD) rats.

**Methods:** Aortic rings (3 mm in length) respectively isolated from 16-week-old SD rats were placed in organ baths with carbogen. Cumulative concentration–relaxation curves (CCRC) to bisoprolol and atenolol (10^-8^-10^-5^mol/L) were evaluated by isometric force recording, after rings were contracted to a plateau by phenylephrine（PE） 10^-6^ mol/L. The effect of bisoprolol was also obtained in the presence of N^ω^-nitro-L-arginine methyl ester (L-NAME, 10^-4^mol/L, an endothelial nitric oxide synthase (eNOS) inhibitor). Vasodilations to acetylcholine (10^-6^ mol/L) greater than 70% were regarded as endothelium-intact vessels, whereas lack of relaxation was taken as proof of endothelium-denuded.

**Results:** In endothelium-intact aortic rings from both strains, bisoprolol induced concentration-dependent vasorelaxations, which showed significant difference with atenolol groups (*P*=0.013). After endothelium removal, the vasodilation response to bisoprolol was inhibited (*P*=0.001). Similarly to the impaired endothelium -independent relaxation, the vasorelaxation effect of bisoprolol was significantly attenuated during incubation of L-NAME in endothelium-intact aortic rings (*P*=0.882).

**Conclusion:** These results suggest that bisoprolol, but not atenolol, exert their vasodilatory action through the NO pathway， and that treatment may be of benefit in conditions of increased central aortic pressure in hypertension.

【Key Words】bisoprolol; atenolol; eNOS; aortic vasorelaxation

β-blocking drugs might have heterogeneouse effects on the arterial system depending on their pharmacologic properties, no specific study was performed to investigate selective β1 blockade bisoprolol. Our objective was to determine whether or not bisoprolol and atenolol induced vasodilation, the relaxant efficacy of vascular rings from SD rats was evaluated by isometric force recording.

**Methods**

Animal preparation and vessel preparation

The experiments were carried out on 16-week-old male Sprague-Dawley (SD) rats. Animals were anaesthesized with pentobarbital (30 mg/kg, i.p.) and exsanguinated. Then, thoracic aortae were dissected immediately and carefully cleaned of fat and connective tissues and cut into rings 3 mm in length. Aortic rings from rats were suspended on stainless steel wires at 37^。^C in a 5-ml organ bath containing Krebs solution composed as follows (mM):118.3 NaCl, 4.7 KCl, 1.2 MgSO4, 1.2 KH2 PO4, 20NaHCO3, 0.016 EDTA, 11.1 glucose, and 2.5 CaCl2 (pH 7.4). Rings were bubbled continuously with carbogen (95%O2/5% CO2) and were equilibrated for 1 h at a resting tension of 2g. Isometric tension was recorded by a force displacement transducer (Danish Myo Tecnology Model 610M, Denmark) in 5 mL organ bath, aerated with 95% O2 and 5% CO2 under an initial resting tension of 19.6mN. Force was recorded via a PowerLab/8sp data acquisition system (A.D. Instruments, Castle Hill, Australia).

Measurement of isometric force in vascular rings

At the beginning of each experiment, ring segments were depolarized with 70 mM potassium chloride (KCl) to determine maximal contractile capacity of the vessel. Rings were then thoroughly washed and allowed to equilibrate.

Cumulative concentration-relaxation curves (CCRC) to bisoprolol and atenolol (10^-8^-10^-5^mol/L) were evaluated by isometric force recording, after the rings were contracted to a plateau with phenylephrine (PE; 10^-6^ mol/L). The effect of bisoprolol was also obtained in the presence of N^ω^-nitro-L-arginine methyl ester (L-NAME; 10^-4^ mol/L), an endothelial nitric oxide synthase (eNOS) inhibitor. Vessels showing a vasodilation greater than 70% in response to acetylcholine (10^-6^ mol/L) were regarded as endothelium-intact vessels, whereas lack of relaxation was taken as a proof of endothelial denudation [1].

**Data analysis**

In vitro studies, statistical significance was estimated by Student t-test or one-way analysis of variance (ANOVA) followed by the Newman-Keuls test. A *P*-value of ≤0.05 (two-sided) was considered statistically significant.

**Results**

As shown in Figure 4, bisoprolol induced concentration-dependent vasorelaxation in endothelium-intact aortic rings, which was significantly higher than that in the atenolol group (*P*=0.013). Furthermore, after endothelium removal, the vasodilatory response to bisoprolol was inhibited (*P*=0.001). Similar to the impaired endothelium-independent relaxation, the vasorelaxant effect of bisoprolol in endothelium-intact aortic rings was significantly attenuated after incubation with L-NAME (*P*=0.882).

**Discussion**

Our experimental evidence demonstrated that bisoprolol, but not atenolol, could produce vasorelaxation on PE pre-contracted aorta rings in rats. Bisoprolol’s vasodilating effects depend on endothelium-dependent mechanisms as inferred from their attenuation by nitric oxide synthase (NOS) inhibitors: N^ω^-nitro-L-arginine methyl ester (L-NAME).

The endothelium-dependent vasodilator effects of bisoprolol may contribute to the decrease in CAP in hypertensive patients. Further studies to elucidate the underlying mechanism are yet to be undertaken.

**References**

Yeh JL, Liou SF, Liang JC, Lee CH, Chiu CC, Lin YT, Chen IJ. Labedipinedilol-C: a third-generation dihydropyridine-type calcium channel antagonist displaying K+ channel opening, NO-dependent and adrenergic antagonist activities. *J Cardiovasc Pharmacol* 2005; 46:130-140.

**Figure legends**

**Figure S1** Concentration–relaxation-response curves to bisoprolol and atenolol exhibited by endothelium-intact aorta artery rings obtained from Sprague-Dawley (SD) rats precontracted with phenylephrine (PE) 10^-6^ mol/L. Endo+: endothelium-intact aortic rings; endo-: endothelium -denuded aortic rings; or endothelium-intact aortic rings in the presence of N^ω^-nitro-L-arginine methyl ester (L-NAME, 10^-4^mol/L). Results are expressed as the percentage of relaxation from the maximal contraction level. Each point is the mean of three separate experiments. **P* < 0.05
