# Supplementary material for: A Randomized Controlled Study on the Effects of Bisoprolol and Atenolol on Sympathetic Nervous Activity and Central Aortic Pressure in Patients with Essential Hypertension
Source: PLoS One. 2013 Sep 10;8(9):e72102. doi: 10.1371/journal.pone.0072102 (PMC3769307; doi:10.1371/journal.pone.0072102)
Supplement: Protocol S1 — Trial Protocol. (PDF) [file pone.0072102.s006.pdf]

Clinical Trial Protocol

**Title**	Effects of bisoprolol and atenolol on resting heart rate, sympathetic nervous system's activity and central blood pressure in patients with essential hypertension（Biso-ARTS Study）
**Phase**	IV
**Sponsor**	Shanghai Ruijin Hospital
**Protocol Version**	Version 5.0/21 March 2011

Princeple Investigator

SIGNATURE PAGE

Title： Effects of bisoprolol and atenolol on resting heart rate, sympathetic nervous system's activity and central blood pressure in patients with essential hypertension（Biso-ARTS Study）

Princeple Investigator

Professor Pingjin Gao

Ruijin Hospital

No.197,Rui Jin Er Road,Shanghai 200025 China

I, the undersigned, am responsible for design and conduct of the study at this site and affirm that:

I understand and will conduct the study according to the protocol, any approved protocol amendments, and all applicable Health Authority requirements and national laws.

I will not deviate from the protocol without prior written permission from the Sponsor, except where necessary to prevent immediate danger to the subject.

____________________________________ ____________________________

Signature *Date of Signature*

Insert Name, academic qualifications

Insert Position (job title)

Insert Address of Institution

Insert Phone, fax, e-mail

List of Abbreviations

AE	Adverse Event
CRF	Case Report Form
SAE	Serious Adverse Event
BRS	Baroreflex Sensitivity
SNS	Sympathetic Nervous System
HRV	Heart Rate Variability
RHR	Resting Heart Rate
BPV	Blood Pressure Variability
EH	Essential Hypertension
PP	Per Protocol
ITT	Intention to Treat

1. Synopsis

**Study title**	Effects of bisoprolol and atenolol on resting heart rate, sympathetic nervous system's activity and central blood pressure in patients with essential hypertension（Biso-ARTS Study）
**Study center(s)/country(ies)**	1 site/China
**Study Phase**	Phase IV
**Planned study period**	First Patient In: September.2010<br>Database Lock: October.2011
**Study objectives**	Primary objective<br>To compare the efficacy in SNS activity and central blood pressure(CAP) of bisoprolol and atenolol in essential hypertensive patients with RHR>70bpm<br>Secondary objective<br>1. To compare the effect in other SNS indication of bisoprolol and atenolol in EH patients with RHR>70bpm<br>2. To compare the efficiency of bisoprolol and atenolol to attaining RHR<65bpm]<br>3. To compare the efficiency of bisoprolol and atenolol on peripheral blood pressure<br>4. To compare the safety and compliance of bisoprolol and atenolol treatment after RHR<65bpm
**Study design and plan**	A phase IV single-centre randomize controlled study. The patient will receive bisoprolol 5mg Qd.(or atenolol 25mg Bid) for 2 weeks. If his/her heart rate<65bpm, this initial dose will be prolonged for another 2 weeks before the observation is over.<br>If the heart rate>65bpm, the patient will receive bisoprolol 7.5mg Qd.(or atenolol 37.5mg Bid) for 2 weeks. Then if his/her heart rate<65bpm, this

	increased dose will be prolonged for another 2 weeks before the observation is over.<br><br>If the heart rate>65bpm, the patient will receive bisoprolol 10mg Qd.(or atenolol 50mg Bid) for 2 weeks. Then if his/her heart rate<65bpm, this increased dose will be prolonged for another 2 weeks before the observation is over. If the heart rate>65bpm, the observation is over directly.
**Planned number of subjects**	110patients
**Diagnosis and main inclusion and exclusion criteria**	Main Inclusion Criteria including:<br>1. 25~65 years old<br>2. Untreated mild to mederate EH<br>3. Sinus rhythm<br>4. RHR ＞70bpm<br>5. Can give written informed consent<br><br>Main Exclusion including including:<br>1. Atrial Fibrillation (AF)/ Sick Sinus Syndrome (SSS)/ atrioventricular block 2-3 grade(AVBⅡ Ⅲ) without pacemaker<br>2. Bradyrrhythmia<br>3. hypotensive<br>4. Unstable Angina Pectoris (UAP)/AMI/ HF (NYHA class III - IV)<br>5. Uncontroled diabetes mellitus (DM)<br>6. Bronchial asthma<br>7. Gastro-intestinal ulcer or skin ulcer<br>8. Liver dysfunction/ renal impairment<br>9. Treated with CCB (Calcium antagonists) (except amlodipine) or other beta blocker.<br>10. Glaucoma<br>11. Known allergic/ intolerance to beta blocker<br>12. Pregnant or lactating women<br>13. Participation in another clinical study within th last 3 months<br>14. Legal incapacity or limited legal capacity
**Study drug**	bisoprolol 5mg<br><br>atenolol 25mg/50mg

**Planned duration of observation for each subject**	1 month at least; 2 months at most

2. Trial administrative structure

2.1 Princeple Investigator

Professor Pingjin Gao
Ruijin Hospital
No.197,Rui Jin Er Road,Shanghai 200025 China

3. Background

The sympathetic nervous system (SNS) plays a role in the pathophysiology of chronic arterial hypertension by modifying cardiac output and peripheral vascular resistance [1,2]. It is known that sympathetic nerve activity (SNA) can cause changes in blood pressure (BP) through the activation of baroreceptors [3,4]. Although introduced into scientific practice, methods of SNA evaluation are not commonly used in a clinical setting. Analysis of baroreflex sensitivity (BRS) and heart rate variability (HRV) have been recommended as the diagnostic tools for evaluating SNA and can be found in clinical guidelines as basic assessment methods [5-7]. Data suggest that low BRS and/or HRV are risk factors for cardiovascular morbidity and mortality [8-10].

Increasing clinical evidence suggests that central aortic pressure (CAP), but not brachial BP, predicts cardiovascular events, because the left ventricle (LV) pumps directly against the afterload in the central arteries. Moreover, aortic systolic BP, pulse pressure (PP), and augmentation index (AIx) have been shown to be strong independent cardiovascular risk predictors in hypertensive populations [11-15].

β-blockers (BBs) are believed to improve SNS function; however, clinical studies on the effects of BBs on HRV and/or BRS in hypertensive patients have shown mixed results [16,17]. Moreover, it is noteworthy that in a series of studies [14,18-22]. atenolol-based therapy was significantly less effective in lowering aortic systolic and pulse pressure than other hypertensive classes， which may be attributed to a different mechanism of atenolol, thus explaining the different clinical outcomes. Since β-blocking drugs might have heterogeneous effects on the arterial system and BRS depending on their pharmacologic properties, further comparisons of the effects of BBs on the arterial system and BRS may be helpful [23]. Bisoprolol with its high β1-selectivity, long duration of action, and favorable

pharmacokinetic properties was shown to be an effective and safe antihypertensive agent [24,25]. Supposedly, these properties of bisoprolol should be an advantage in clinical practice.

Therefore, the present study was designed to compare the effects of a highly selective β1-blocker (bisoprolol) and a classical β-blocker (atenolol) on SNA and CAP in hypertensive patients with controlled heart rate.

4. Study Objectives

Primary objective

To compare the efficacy in SNS activity and central blood pressure(CAP) of bisoprolol and atenolol in essential hypertensive patients with RHR>70bpm

Secondary objective

1. To compare the effect in other SNS indication of bisoprolol and atenolol in EH patients with RHR>70bpm
2. To compare the efficiency of bisoprolol and atenolol to attaining RHR<65bpm]
3. To compare the efficiency of bisoprolol and atenolol on peripheral blood pressure

To compare the safety and compliance of bisoprolol and atenolol treatment after RHR<65bpm

4.1 Primary objective

To compare the efficacy in SNS activity and central blood pressure(CAP) of bisoprolol and atenolol in essential hypertensive patients with RHR>70bpm

4.2 Secondary objective

1. To compare the effect in other SNS indication of bisoprolol and atenolol in EH patients with RHR>70bpm
2. To compare the efficiency of bisoprolol and atenolol to attaining RHR<65bpm]
3. To compare the efficiency of bisoprolol and atenolol on peripheral blood pressure

5. Study Design

5.1 Study Design

It is a single-centers, randomized study in China, and planed to enroll 110 essential hypertension patients from 1 sites, who will be assigned to two groups randomly. All the inclusive patients will be consistent with the diagnosis of essential hypertension, and have a resting heart rate(RHR) ≥70 beats per minutes(bpm).

The inclusive patients will be assigned to two groups as follow (ratio 1:1)

Arm A: bisoprolol, 5-10mg/day for a targeting heart rate of less than 65 bpm(96)

Arm B: atenolol, 50-100mg/day for a targeting heart rate of less than 65 bpm (96)

The patient will receive bisoprolol 5mg Qd.(or atenolol 25mg Bid) for 2 weeks. If his/her heart rate<65bpm, this initial dose will be prolonged for another 2 weeks before the observation is over.

If the heart rate>65bpm, the patient will receive bisoprolol 7.5mg Qd.(or atenolol 37.5mg Bid) for 2 weeks. Then if his/her heart rate<65bpm, this increased dose will be prolonged for another 2 weeks before the observation is over.

If the heart rate>65bpm, the patient will receive bisoprolol 10mg Qd.(or atenolol 50mg Bid) for 2 weeks. Then if his/her heart rate<65bpm, this increased dose will be prolonged for another 2 weeks before the observation is over. If the heart rate>65bpm, the observation is over directly.

5.1.1 Study flow

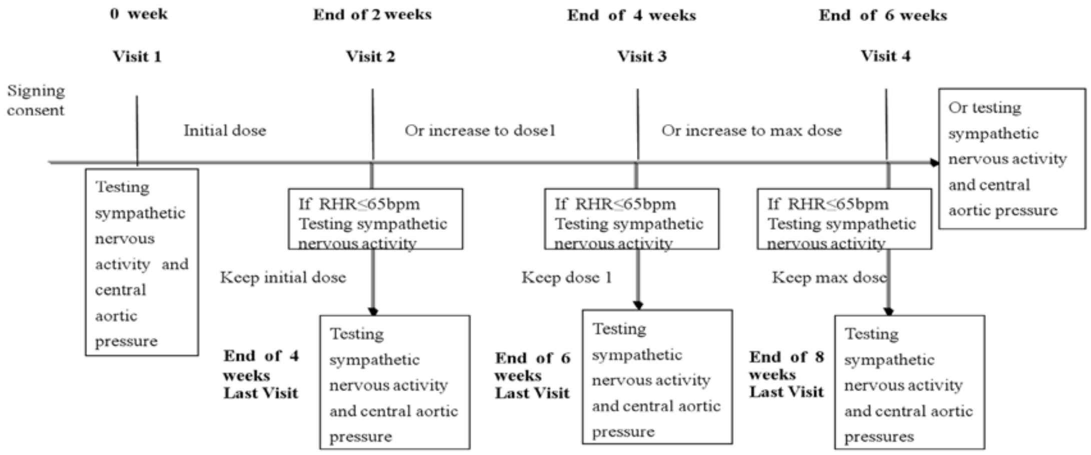

5.1.3 Dosage adjustment

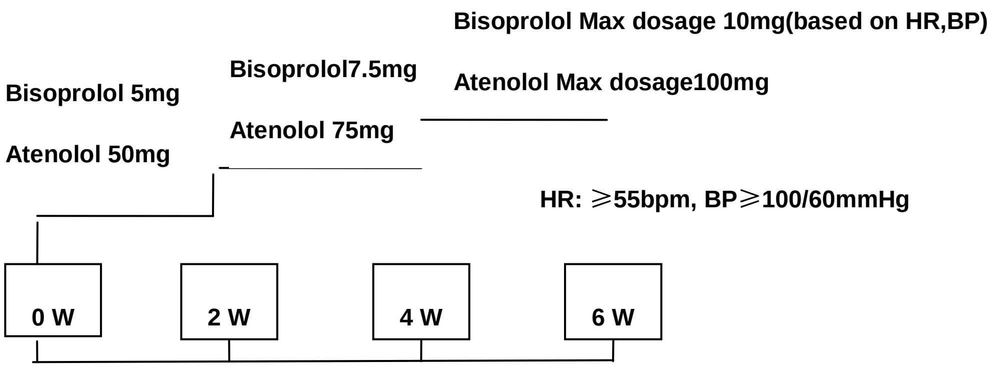

5.2 Trial design

5.2.1 Inclusion of Special Populations

This study will not include special populations such as children, pregnant women and subjects with genetic diseases.

5.3 Selection of Trial Population

5.3.1 Subjects enroll

All patients who meet the inclusion criteria but not the exclusion criteria can be enrolled into this study.

5.3.2 Inclusion Criteria：

- 25~65 years old
- Untreated mild to mederate EH
- Sinus rhythm
- RHR ＞70bpm
- Can give written informed consent

5.3.3 Exclusion Criteria:

- Atrial Fibrillation (AF)/ Sick Sinus Syndrome (SSS)/ atrioventricular block 2-3 grade(AVBII -III) without pacemaker
- Bradyrrhythmia
- hypotensive
- Unstable Angina Pectoris (UAP)/AMI/ HF (NYHA class III - IV)
- Uncontroled diabetes mellitus (DM)
- Bronchial asthma
- Gastro-intestinal ulcer or skin ulcer
- Liver dysfunction/ renal impairment
- Treated with CCB (Calcium antagonists) (except amlodipine) or other beta blocker.
- Glaucoma
- Known allergic/ intolerance to beta blocker
- Pregnant or lactating women
- Participation in another clinical study within the last 3 months
- Legal incapacity or limited legal capacity

5.3.4 Randomization:

This study is a parallel, randomized, controlled study, Patient will be randomized by the predesigned randomization schedule using randomization envelop.

5.3.5 Criteria for Subject Withdrawal

Subjects are free to discontinue the trial at any time without giving their reasons.

A subject must be withdrawn in the event of any of the following:

1. Withdrawal of the subject’s consent.

2. Occurrence of an exclusion criterion which is clinically relevant and affects the subject’s safety, if discontinuation is considered necessary by the Investigator.

3. Intolerance to Concor® treatment.

4. Occurrence of unexpected adverse events that are unacceptable in nature, severity and/or duration, or the frequency of known unacceptable adverse events is higher than expected.

5.3.6 Premature Discontinuation of the Trial

The trial may be stopped by the Investigator in the event of any of the following:

1. Medical or ethical reasons that hinder the continuation of the study.

2. Poor enrollment of subjects

3. Occurrence of unexpected adverse events that are unacceptable in nature, severity and/or duration, or the frequency of known unacceptable adverse events is higher than expected.

The whole trial may be temporarily suspended or permanently terminated. If premature discontinuation of the trial is decided, the ethics committee will be informed in accordance with applicable regulations.

6. Study Medicine

6.1 Medication

Bisoprolol should be taken in morning orally.

Atenolol should be taken in morning orally.

6.2 Dosage

Intial period（2 weeks）： Bisoprolol5mg qd or Atenolol50mg qd

Fisrt dosage adjustment period（2 weeks）：Bisoprolol 7.5mg qd or Atenolol 75mg qd

Second dosage adjustment period（2 weeks）：Bisoprolol 7.5mg qd or Atenolol 75mg qd

Extended treatment period(2 weeks): When patient’ s heart rate is lower than 65 bpm, keep the dosage for 2 more week.

6.3 Concomitant Medications and Therapies

During the trial, any concomitant drugs, such as anti-diabetic drugs, lipid lowering drugs, anticoagulants and heart failure treatments like diuretics and ACEI, may be continued provided that these drugs do not affect heart rate. All medications used 3 months before enrollment and during the entire trial should be recorded. The dosage of these drugs may be adjusted as long as there is no effect on the heart rate.

6.3 Non-permitted Medicines

CCB, ACEI, ARB, diuretics and β-blockers other than Concor® are not permitted during the trial.

6.4 Specification, Packaging and special precautions

Product: Bisoprolol

Specification: 5mg

Packaging: plastic packaging, 10 tablets/ box

Producer: Merck Serono, Darmstadt, Germany

Note: stored at below 30°C. Keep out of reach of children. Expired drug should not be used

Product: Atenolol

Specification: 25mg/ 50mg

Packaging: plastic packaging, 100tablets/ box

Producer: Beijing Double-Crane Pharmaceutical Co., Ltd, Beijing, China

Note: stored at below 25°C. Keep out of reach of children. Expired drug should not be used

6.5 Treatment of Overdose

The common drug overdose reactions of β-adrenergic receptor blockers include bradycardia, hypotension, bronchial asthma, acute heart failure and low blood sugar. There are only a small number of oral drug overdose reports on Concor ® (maximal dose of 2000mg), in which bradycardia and/or low blood pressure occurs and all patients recovered after withdrawn of the drug.

If an overdose occurs, the patient should be hospitalized for observation and proper supportive treatment should be administrated.

Any overdose must be recorded in the CRF and reported to the Sponsor and relevant authorities.

7.1 Trial procedures and assessments

7.1 Trial schedule and assessment contents

The trial procedure and assessment contents are as follows:

1. Visit 1 -Week -1 (1 +3 day): this visit entails the following:

Before any procedures that are related to the trial is undertaken, informed consent of the patient should be obtained. Thereafter, basic information of the patient will be obtained and patients are screened for eligibility based on the inclusion and exclusion criteria.

①. Record birth date, gender, height, weight, waist circumstance, current medical condition and past medical history, including concomitant therapies.

②. Perform physical examinations, including vital signs (blood pressure, heart rate).

③. Blood tests: full blood count (white blood cells, neutrophils, red blood cells, hemoglobin and platelets), liver function (ALT, AST), renal function (Bun, Cr), fasting blood glucose and blood lipids (TC, TG, LDL-C and HDL-C).

④. 12-Lead ECG and echocardiography

⑤. Sepical tests: central blood pressure, BRS, BPV, HRV, Holter and 24 h-ABPM

2. Visit 2-Week 2 (14+3 days): this visit entails the following:

①. Perform physical examinations, including vital signs (blood pressure, heart rate), 12-Lead ECG.

②. If the RHR is lower than 65 bpm, test BRS, BPV, HRV. Remain the treatment for an extended 2-week, then go for Last visit.

③. Record AE and concomitant therapies.

3. Visit 3-Week 4 (28 +3 days): this visit entails the following:

①. Perform physical examinations, including vital signs (blood pressure, heart rate), 12-Lead ECG.

②. If the RHR is lower than 65 bpm, test BRS, BPV, HRV. Remain the treatment for an extended 2-week, then go for Last visit.

③. If the RHR is still higher than 65 bpm, adjust the dosage of bisoprolol or atenol according to blood pressure and heart rate.After 2 weeks, go for Visit 4.

④. Record AE and concomitant therapies.

4. Visit 4-Week 6 (42+3 days): this visit entails the following:

①. Perform physical examinations, including vital signs (blood pressure, heart rate), 12-Lead ECG.

②. If the RHR is lower than 65 bpm, test BRS, BPV, HRV. Remain the treatment for an extended 2-week, then go for Last visit.

③. If the RHR is still higher than 65 bpm, directly go for Last visit.

④. Record AE and concomitant therapies.

5. Last Visit- End of extended treatment:

①. Perform physical examinations, including vital signs (blood pressure, heart rate), 12-Lead ECG.

②. Blood tests: full blood count (white blood cells, neutrophils, red blood cells, hemoglobin and platelets), liver function (ALT, AST), renal function (Bun, Cr), fasting blood glucose and blood lipids (TC, TG, LDL-C and HDL-C).

③. Sepical tests: central blood pressure, BRS, BPV, HRV, Holter and 24 h-ABPM

④. Record AE and concomitant therapies.

7.2 Demographic and Other Baseline Characteristics

7.2.1 Demographic Statistics

Major demographic information is as follows:

1. Name initials
2. Birth date
3. Gender
4. Race

7.2.2 Informed Consent

Participants must sign a written informed consent.

7.2.3 Medical history

The following information will be collected for the medical history of the subjects:

1. To confirm whether the subjects meet the inclusion or exclusion criteria
2. Past and current medical history, smoker should be recorded smoking history.
3. Past therapies and current concomitant drugs.

7.2.4 Vital signs and physical examination

Vital signs and physical examination should at least include the following items:

1. General status:
   - ✓ Heart rate (resting heart rate) measurement: sit rest for 5 minutes, no smoking, no excitatory food and beverage such as tea and coffee for 30 minutes, measurements are taken at sitting position for a continuous record of 3 minutes. Heartbeats in each minute are calculated and averaged to obtain the resting heart rate.
   - ✓ Blood pressure measurement: sit rest for 5 minutes, no smoking, no excitatory food and beverage such as tea and coffee for 30 minutes, measurements are taken at sitting position, with the elbow at the same level with the heart. Diastolic blood pressure is recorded at the fifth Korotkoff sound, repeated after 2 minutes and averaged. If the difference in diastolic blood pressure readings is > 5mmHg, measurement is repeated again after 2 minutes, and the 3 readings are averaged.
2. Physical examination of the respiratory system

3. Physical examination of the cardiovascular system

4. Physical examination of other systems

5. Body mass index: body weight (Kg)/Height ^{2}(m^2)

6. Measurement of waist circumstance: At screening visit, under fasting, standing, calm breathing conditions, feet apart 25-30cm, waist circumstance is measured at horizontal position at the midpoint of iliac spine and the 12th rib, with measuring tape close to but not press the skin, accurate to 0.1cm.

7.2.5 Lab test items

1. Full blood count: red blood cell count, neutrophils, hemoglobin, white blood cell count and platelet count

2. Urine test

3. Liver function (ALT, AST, ALP)

4. kidney function (BUN, Cr)

5. Chemistry tests: Na, K, Cl

6. fasting blood glucose

7. HbA 1c

8. Blood lipid levels (TC, TG, HDL-C, LDL-C)

9. 12-Lead ECG

10. echocardiography: to measure ejection fraction (EF%), fractional shortening (Fs), left ventricular end systolic diameter (LVESD), interventricular septum thickness (IVST), left ventricular posterior wall thickness (PWT)

7.2.6 Special Tests

11. Central blood pressure

    Central blood pressure was performed by pressure tonometry using the integral software (SphygmoCor; AtCor Medical, Sydney, Australia) at the radial pulse, considering that this system has shown good repeatability of measurements. AIx, a measure of systemic arterial stiffness, was calculated as the difference between the second and first systolic peaks, expressed as a percentage of the pulse pressure. Because AIx depends on heart rate (HR), it was corrected for a HR of 75 bpm (AIxatHR75)

12. BRS, BPV and HRV

Measurements of SNA were performed after a 30-min rest in the sitting position. Patients were monitored noninvasively with a Finometer (Finapres Medical Systems, Amsterdam, The Netherlands; factory number: FMI. MU 00694, 00637; operation parameters: input: 220–240 V, 50–60 Hz, 100 VA) in both the supine (henceforth referred to as 'lying') and the standing positions. BRS was defined by the slope of the linear regression curve obtained by plotting the changes of SBP against the pulse interval. The mean value of the various slopes was calculated and used as the definitive BRS value for each subject [5,30-33]. HRV was calculated from the time-sequential analysis and was expressed as 3 components: low frequency (LF), high frequency (HF), and LF/HF ratio.

13. 24-hour ABPM

    Blood pressure variability (BPV) was also calculated in ABPM

14. 24-hour Holter

    For dectect cardiac arrhythmia.

7.3 Assessment of Efficacy

Assessment of efficacy mainly includes the following items:

1. Heart rate
2. Blood pressure, including peripheral or central blood pressure.
3. BRS
4. BPV
5. HRV

7.4 Assessment of Safety

7.4.1 Management and reporting of adverse events

7.4.1.1 Definitions

Adverse Event

An adverse event (AE) is any untoward medical occurrence in a subject or clinical investigation subject administered a pharmaceutical product, which does not necessarily have a causal relationship with this treatment.

An AE can therefore be any unfavorable and unintended sign (including an abnormal laboratory finding), symptom, or disease temporally associated with the use of a medicinal product, whether or not considered related to the medicinal product.

Investigators must assess the severity/intensity of adverse events according to the Qualitative Toxicity Scale as follows:

Mild: The subject is aware of the event or symptom, but the event or symptom is easily tolerated.

Moderate: The subject experiences sufficient discomfort to interfere with or reduce his or her usual level of activity.

Severe: Significant impairment of functioning: the subject is unable to carry out usual activities.

Investigators must also systematically assess the causal relationship of AEs to Concor® using the following definitions. Decisive factors for the assessment of causal relationship of an AE to the treatments include, but may not be limited to, temporal relationship between the AE and the treatments; known side effects of treatments; concomitant medication, course of the underlying disease or impact of other treatment.

Serious Adverse Event

A serious adverse event (SAE) is any untoward medical occurrence that at any dose:

- Results in death.
- Is life-threatening.

NOTE: The term “life-threatening” in this definition refers to an event in which the subject is at risk of death at the time of the event; it does not refer to an event that hypothetically might cause death if it were more severe.

- Requires inpatient hospitalization or prolongation of existing hospitalization.
- Results in persistent or significant disability/incapacity.
- Is a congenital anomaly/birth defect.
- Is otherwise considered as medically important.

Important medical events that may not result in death, be life-threatening, or require hospitalization may be considered as SAEs when, based upon appropriate medical judgment, they may jeopardize the subject or may require medical or surgical intervention to prevent one of the outcomes listed in this definition. Examples of such events include allergic bronchospasm requiring intensive treatment in an emergency room or at home, blood dyscrasias or convulsions that do not result in inpatient hospitalization.

Relevant serious adverse events that need to be intensively monitored in this trial:

1. Ⅲ Degree atrioventricular block;

2. Severe asthma attack;
3. Cardiac arrest
4. Acute pulmonary edema;
5. Worsening heart failure;
6. Shock.

Events that Do Not Meet the Definition of an SAE

Elective hospitalizations to simplify study treatment or study procedures are not considered an SAE. However, all events leading to unplanned hospitalizations or unplanned prolongation of an elective hospitalization must be documented and reported as SAEs.

7.4.1.2 Recording Adverse Events

All adverse eventsoccurring during the study, whether serious or not, must be recorded in the Case Report Form (CRF), including its description, severity, duration (onset and resolution dates), causal relationship (i.e., whether the adverse event is related to the study treatment), any other potential causal factors, actions taken with the study drug (dose reduction, withdrawal…), required treatment, and outcome. In addition, for all serious adverse events, seriousness criteria must be documented and a specific safety report form must be completed.

7.4.1.3 Expedited Reporting of Serious Adverse Events

As soon as the Investigator detects a serious adverse event, as defined above, associated with the study medication, he/she will report it within 24 hours (by e-mail, phone, or fax) to princepal investigator Gao Pingjin.

The Investigator must promptly respond to any request for follow-up information or questions from the Sponsor. Serious adverse events occurring during the study must be monitored and followed up by the Investigator until stabilization or until the outcome is known, unless the subject is lost to follow-up. The Investigator will ensure any necessary additional therapeutic measures and follow-up procedures.

Reporting procedures and timelines for any new information on a previously reported serious adverse event (follow-up) are the same as for the original report.

Further to reporting serious adverse events to the Sponsor, the Investigator will comply with any applicable local pharmacovigilance requirements to report adverse reactions/events to national pharmacovigilance systems.

7.4.3 Other laboratory tests

During the study period, laboratory tests other than these specified in the protocol will be

decided based on the needs of clinical conditions.

8 Statistics

8.1 Sample Size

This study will recruit 110 patients with RHR≥ 70bpm and mild or moderate blood pressure.

8.2 Randomization

Patient will be randomized in a 1:1ratio.

8.2 Randomization

All subjects will be treated according to the dose approved for their condition, and the subsequent treatment will be carried out according to the standard care and medical practice of the study site. Therefore, no randomization is required for this study.

8.3 Endpoints

8.3.1 Primary Endpoint(s)

The primary endpoint is BRS and central blood pressure at Last visit.

8.3.2 Secondary Endpoint(s)

The secondary endpoints in this study include

①. Effective endpoints:

i. RHR

ii. BPV

iii. Blood pressure: peripheral and central blood pressure

②. Safety endpoints: AEs recoreded in this study

③. Compliance Analysis

8.4 Analysis Sets

8.4.1 PP and ITT Analysis

The main analysis set will be defined as all subjects who receive at least one dose of any investigational medicinal product, i.e., the Intention-to-Treat (ITT) population. This population will be used for all efficacy evaluations. A secondary analysis set, the Per Protocol

(PP) set, will also be defined, which will exclude all subjects found to have a major protocol deviation*.

*Deviation from the protocol will be determined by medical check.

8.4.2 Subgroup Analysis

Subgroup analysis will be conducted within the subgroups defined according to drug dosage.

8.4.3 Safety Analysis

The set of subjects included in the safety analysis is those subjects who receive at least one dose of trial drug and who have follow-up safety data.

8.5 Description of Statistical Analyses

8.5.1 General Considerations

All statistical tests will be performed at the 0.05 significance level. 95% confidence intervals will be calculated whenever appropriate.

Only available data will be considered for analysis. Subjects who discontinue the study prematurely will be considered as missing data and will be dropped from the statistical analysis.

9. Ethical and Regulatory Aspects

9.1 Responsibilities of the Investigator

The Investigator is responsible for the conduct of the trial at his/her site. He/she will ensure that the trial is performed in accordance with the Declaration of Helsinki, as well as with the ICH Note for Guidance on Good Clinical Practice (ICH E6, 1996) and applicable regulatory requirements. In particular, the Investigator must ensure that only subjects who have given their informed consent are included into the trial.

9.2 Subject Information and Informed Consent

Implementation of the above item should comply with the ICH-GCP guidelines (ICH，E6，1996), management regulations of the clinical trial and applicable regulations of local hospital.

9.3 Informed Consent

9.3.1 Obtaining informed consent

Written consent form for participation of the clinical trial should be presented to the subject before any relevant tests. Informed consent must be signed and dated by the subjects and investigators, or these who assigned by the investigator to serve as interpreters of the

informed consent.

Detailed procedure should comply with the ICH-GCP guidelines (ICH，E6，1996), management regulations of the clinical trial and applicable regulations of local hospital.

Subjects and investigators must sign and date the informed consent. Signed and dated informed consent forms should be kept by the investigators and properly archived for inspection or audit at any time. The subjects should be given a copy of the informed consent.

9.3.2 Case the subject cannot complete the informed consent by him/herself

9.3.2.1 Illiterate subjects

If the subject or their legal representative is illiterate, then the entire informed consent process should have an independent witness present. Choice of witnesses should not be contrary to the confidentiality right of the subject. A reliable and independent witnesses should be not affiliated with the research institutions or investigators involved in the study. Family members or acquaintances of the subject are suitable candidates for the independent witness. Upon oral agreement, the subject or their legal representative should sign if capable, and the witness should also sign and date the informed consent form to confirm that the information is accurate and the subject or their legal representative completely understands the contents of the informed consent, i.e., a true informed consent.

9.4 Subjects Insurance

Compensation for the subject should comply with state laws, the GCP rule, terms of the insurance company and relevant regulations of the hospitals participating in the study.

9.5 Ethics Committee or Institutional Review Board

Prior to commencement of the trial, the trial protocol will be submitted together with its associated documents (subject information, consent form, investigator handbook) to the responsible Independent Ethics Committee (IEC)/Institutional Review Board (IRB) for its favorable opinion/approval. The written favorable opinion/approval of the IEC/IRB must be obtained before initiation of the trial.

10. Trial Management

10.1 Case Report Form Handling

The main purpose of the Case Report Form (CRF) is to obtain those data required by the trial protocol in a complete, accurate, legible and timely fashion. The data in the CRF should be consistent with the relevant source documents.

Detailed procedure should comply with the ICH-GCP guidelines, management regulations of the clinical trial and applicable regulations of local hospital.

10.2 Source Data and Subject Files

Detailed procedure should comply with the ICH-GCP guidelines, management regulations of the clinical trial and applicable regulations of local hospital.

The Investigator must keep a subject file (medical file, original medical records) on paper or electronically for every subject included in the trial. This file will contain the available demographic and medical information for the subject, and should be as complete as possible. In particular, the following data should be available in this file:

1. Subject’s full name,
2. Date of birth,
3. Gender,
4. Medical history and concomitant diseases,
5. Concomitant therapies (including changes during the trial),
6. Date of subject’s inclusion into the trial (i.e. date of giving informed consent),
7. Dates of the subject’s visits to the site,
8. Any medical examinations and clinical findings predefined in the protocol,
9. All adverse events observed in the subject,
10. Date of subject’s end of trial, and
11. Date of and reason for early withdrawal of the subject from the trial or from Investigational Medicinal Product, if applicable.

10.3 Investigator Site File and Archiving

Detailed procedure should comply with the ICH-GCP guidelines, management regulations of the clinical trial and applicable regulations of local hospital.

10.4 Monitoring, Quality Assurance and Inspection by Authorities

Detailed procedure should comply with the ICH-GCP guidelines, management regulations of the clinical trial, sponsor and applicable regulations of local hospital.

10.5 Changes to the Protocol

Detailed procedure should comply with the ICH-GCP guidelines, management regulations of the clinical trial, sponsor and applicable regulations of local hospital.

10.6 Clinical Trial Report and Publication Policy

10.6.1 Clinical Trial Report

After conclusion of the trial, a clinical trial report will be written by the Sponsor in

consultation with the Coordinating Investigator and other relevant committees or groups.

The first author of the publication shall be the Coordinating Investigator or other investigators designated by the Coordinating Investigator.

10.6.2 Publication

The first publication will be a publication of the results of the analysis of the primary endpoint(s) that will include data from all trial sites.

The Investigator will inform the Sponsor in advance about any plans to publish or present data from the trial. Any publications and presentations of the results (abstracts in journals or newspapers, oral presentations, etc.), either in whole or in part, by Investigators or their representatives will require pre-submission review and written approval of the Sponsor.

The Sponsor will not suppress or veto publications, but maintains the right to delay publication for 3 months in order to protect intellectual property rights.

Reference

1. Julius S, Schork N, Schork A (1988) Sympathetic hyperactivity in early stages of hypertension: the Ann Arbor data set. J Cardiovasc Pharmacol 12 Suppl 3: S121-129.

2. Smith PA, Graham LN, Mackintosh AF, Stoker JB, Mary DA (2004) Relationship between central sympathetic activity and stages of human hypertension. Am J Hypertens 17(3): 217-222.

3. Aksamit TR, Floras JS, Victor RG, Aylward PE (1987) Paroxysmal hypertension due to sinoaortic baroreceptor denervation in humans. Hypertension 9(3): 309-314.

4. Liu HK, Guild SJ, Ringwood JV, Barrett CJ, Leonard BL, et al. (2002) Dynamic baroreflex control of blood pressure: influence of the heart vs. peripheral resistance. Am J Physiol Regul Integr Comp Physiol 283(2): R533-542.

5. Imholz BP, Wieling W, van Montfrans GA, Wesseling KH (1998) Fifteen years experience with finger arterial pressure monitoring: assessment of the technology. Cardiovasc Res 38(3): 605-616.

6. (1996) Heart rate variability: standards of measurement, physiological interpretation and clinical use. Task Force of the European Society of Cardiology and the North American Society of Pacing and Electrophysiology. Circulation 93(5): 1043-1065.

7. Parati G, Di Rienzo M, Mancia G (2000) How to measure baroreflex sensitivity: from the cardiovascular laboratory to daily life. J Hypertens 18(1): 7-19.

8. Vaishnav S, Stevenson R, Marchant B, Lagi K, Ranjadayalan K, et al. (1994) Relation between heart rate variability early after acute myocardial infarction and long-term mortality. Am J Cardiol 73(9): 653-657.

9. Kleiger RE, Miller JP, Bigger JT, Jr., Moss AJ (1987) Decreased heart rate variability and its association with increased mortality after acute myocardial infarction. Am J Cardiol 59(4): 256-262.

10. La Rovere MT, Pinna GD, Hohnloser SH, Marcus FI, Mortara A, et al. (2001) Baroreflex sensitivity and heart rate variability in the identification of patients at risk for life-threatening arrhythmias: implications for clinical trials. Circulation 103(16): 2072-2077.

11. Safar ME, Blacher J, Pannier B, Guerin AP, Marchais SJ, et al. (2002) Central pulse pressure and mortality in end-stage renal disease. Hypertension 39(3): 735-738.

12. Nurnberger J, Keflioglu-Scheiber A, Opazo Saez AM, Wenzel RR, Philipp T, et al. (2002) Augmentation index is associated with cardiovascular risk. J Hypertens 20(12): 2407-2414.

13. Danchin N, Benetos A, Lopez-Sublet M, Demicheli T, Safar M, et al. (2004) Aortic pulse pressure is related to the presence and extent of coronary artery disease in men undergoing diagnostic coronary angiography: a multicenter study. Am J Hypertens 17(2): 129-133.

14. Williams B, Lacy PS, Thom SM, Cruickshank K, Stanton A, et al. (2006) Differential impact of blood pressure-lowering drugs on central aortic pressure and clinical outcomes: principal results of the Conduit Artery Function Evaluation (CAFE) study. Circulation 113(9): 1213-1225.

15. Roman MJ, Devereux RB, Kizer JR, Lee ET, Galloway JM, et al. (2007) Central pressure more strongly relates to vascular disease and outcome than does brachial pressure: the Strong Heart Study. Hypertension 50(1): 197-203.

16. Chen X, Hassan MO, Jones JV, Sleight P, Floras JS (1999) Baroreflex sensitivity and the blood pressure response to beta-blockade. J Hum Hypertens 13(3): 185-190.

17. Chern CM, Hsu HY, Hu HH, Chen YY, Hsu LC, et al. (2006) Effects of atenolol and losartan on baroreflex sensitivity and heart rate variability in uncomplicated essential hypertension. J Cardiovasc Pharmacol 47(2): 169-174.

18. Dahlof B, Devereux RB, Kjeldsen SE, Julius S, Beevers G, et al. (2002) Cardiovascular morbidity and mortality in the Losartan Intervention For Endpoint reduction in hypertension study (LIFE): a randomised trial against atenolol. Lancet 359(9311): 995-1003.

19. Lindholm LH, Carlberg B, Samuelsson O (2005) Should beta blockers remain first choice in the treatment of primary hypertension? A meta-analysis. Lancet 366(9496): 1545-1553.

20. Carlberg B, Samuelsson O, Lindholm LH (2004) Atenolol in hypertension: is it a wise choice? Lancet 364(9446): 1684-1689.

21. Bradley HA, Wiysonge CS, Volmink JA, Mayosi BM, Opie LH (2006) How strong is the evidence for use of beta-blockers as first-line therapy for hypertension? Systematic review and meta-analysis. J Hypertens 24(11): 2131-2141.

22. Morgan T, Lauri J, Bertram D, Anderson A (2004) Effect of different antihypertensive drug classes on central aortic pressure. Am J Hypertens 17(2): 118-123.

23. Cruickshank JM (2007) Are we misunderstanding beta-blockers. Int J Cardiol 120(1): 10-27.

24. Leopold G, Pabst J, Ungethum W, Buhring KU (1986) Basic pharmacokinetics of bisoprolol, a new highly beta 1-selective adrenoceptor antagonist. J Clin Pharmacol 26(8): 616-621.

25. Leopold G (1986) Balanced pharmacokinetics and metabolism of bisoprolol. J Cardiovasc Pharmacol 8 Suppl 11: S16-20.
